# Intra-pulmonary percussive ventilation in complex respiratory therapy patients with ali/ards in cardiovascular and thoracic surgery

**DOI:** 10.1186/2197-425X-3-S1-A277

**Published:** 2015-10-01

**Authors:** RA Ibadov, AS Arifjanov, NA Strijkov

**Affiliations:** Republican Specialized Center of Surgery Named after Acad. V. Vakhidov, ICU, Tashkent, Uzbekistan

## The Aim

Comparison of the effectiveness of the modes of respiratory support, different by pressure and volume (PCV, VCV) to select the optimal variants for mechanical ventilation in patients with of acute respiratory distress syndrome ALI/ARDS.

## Materials and Methods

The patients included in the study were those after cardiopulmonary bypass surgery, coronary artery bypass grafting, and heart valve replacement surgeries, complicated by ARDS, in ICU RSCS after acad. Vakhidov within 2010-2014. In complex respiratory therapy included intra-pulmonary percussive ventilation (IPV).

In a comparative aspect, to divide two group patients: Group A, n = 28. Used mode of mechanical ventilation was with controlled pressure (PCV), an inverse relationship I:E -1,5:1, Pinsp-20-26 cm of water, Fi02 < 60% “Optimal” PEEP, the rate of Vinsp - 40 - 60 l/min, auto PEEP comprising no more than 50% of total PEEP. Group B, n = 26. Used mode of ventilation comprised ventilation with small Vt and low Pplat ( < 35 smH_2_O), with controlled volume (VCV), Ppeak < 35-40 cm of water, VT 6-8 ml/kg, Fi 02 < 60%, PEEP 8-10 mm Hg, the rate of Vinsp - 40 - 60 l/min. The efficiency measure criterions: Pa02 and Sa02, Pa02/Fi02_,_ Fshunt, p02 (A-a), p02 (a/A), Cst., degree of lung injury by J. Murray.

## Results

Improvement of Pa02/Fi02 in group A than in group B (outcome - 108,7 ± 22,4/112,4 ± 20,2, 5-day-184,8 ± 22,4/140,4 ± 24,2. A/B, respectively). There was a difference in values of p02 (A-a) 170 ± 18/165 ± 20 on the 2^nd^ day, 100 ± 20,4/140 ± 22,6 on the 5^th^ day, 58 ± 24,4/100 ± 22,2 respectively. In the first group transition of ALI into ARDS was observed in 2 cases, in the second group - in 4 cases. Compared to group B, reduction of duration of mechanical ventilation was observed in patients of group A (16 ± 4,6/12 ± 2,6 days, respectively). Results are different between patients treated with and without IPV. PEEP, tidal volume and maximal alveolar pressure all increased with IPV.

## Conclusions

Management of patients with ALI/ARDS in the modes by pressure, with restricted peak inspiratory pressure and tidal volume with prolonged inspiratory time with intra-pulmonary percussive ventilation, prove to be more effective for the correction of hypoxemia, for reducing the negative effects of mechanical ventilation on pulmonary parenchyma, for reducing duration of mechanical ventilation and more favorable in terms of lethality when compared with conventional methods of mechanical ventilation.

